# Patterns of Mortality in the Elderly in Chi Linh, Hai Duong, Vietnam, Period 2004–2012

**DOI:** 10.3934/publichealth.2016.3.615

**Published:** 2016-08-22

**Authors:** Quyen Thi-Tu Bui, Cuong Viet Pham

**Affiliations:** 1 Department of Biostatistics, The Hanoi School of Public Health, Ha Noi, Vietnam; 2 Information Technology Unit, The Hanoi School of Public Health, Ha Noi, Vietnam

**Keywords:** mortality, elderly, pattern, Chililab, Vietnam

## Abstract

**Objective:**

This paper examines the trends of mortality in the elderly people in Chi Linh during period 2004–2012 and identifies a number of factors related to mortality in the elderly.

**Design:**

The longitudinal study method is used. The analyzed data is extracted from database of theDemographic—Epidemiologic Surveillance System (DESS) of Chililab. The data is collected from 7 communes/town of Chi Linh district, Hai Duong province during 2004–2012 with all elderly people. Descriptive statistical analysis and survival analysis using Kaplan Meier survival estimates and Cox regression models were used. The indirect standardization was used to compare between the mortality rate of the elderly in Chi Linh and the rates of those in some reference groups.

**Results:**

Mortality rate in elderly tend to decrease over the period 2004–2012. In all the time, mortality rate in elderly men is higher than that in the elderly women. Specific mortality rates by age groups have increased in both males and females. The increase Age specific mortality rates in males is higher than females. Indirect standardized mortality data for the elderly in 2009 in Chi Linh, Vietnam, Canada, the United States of America (USA) showed that elderly mortality rate in Chililab in 2009 was lower than that in elderly of Vietnam (standardized mortality ratio—SMR of elderly in Chililab is only by 75% in comparison with elderly of Vietnam), and also lower than that in elderly people in the US, and Canada. Cox regression analysis (multivariate models) show that with every 1 year older, the risk of death in elderly men increased by 9% and 12% increase in elderly female, for both men and women general risk increased by 10% (*p* < 0.05). Elderly with higher education levels, elderly with better family economic conditions; elderly living with wife/husband have lower mortality risk than the other counterparts.

**Conclusions:**

The research results suggest some recommendations: Strengthening health care programs for elderly people with low education levels, poor economic conditions, and celibacy groups (the vulnerable groups).

## 1. Introduction

Population aging is one important demographic trend in the 21^st^ century and increased rapidly in developed countries where the population aged 60 years old or over has experienced the fastest pace ever: growing at 3.7 per cent annually between 2010 and 2015 and is estimated to go up by 2.9 per cent annually before 2050 and 0.9 per cent annually between 2050 and 2100, increasing from 554 million people in 2013 to 1.6 billion people in 2050 [1]. However, in developing countries, population aged 60 or over rose at 1 per cent annually before 2050 and 0.11 per cent from 2050 to 2100. The rate of ageing population is associated with various health problems in elderly people. Therefore, research and policies should be enhanced in order to improve the care and quality of life of the elderly [1].

The age-specific mortality rates in developed countries indicated that the mortality rates reduced dramatically in young age groups thanks to medical advancements, higher quality of life and mortality rates increased in old age groups due to aging and the increased proportion of the elderly. The mortality in all age groups in the world during 1970–2010 experienced a gradual reduction [2],[3].

The mortality rate of the elderly aged 60–79 declined by about 40%–43% while that of those aged 80 years old or over climbed down by 25% in this period. In all age groups, the reduction rate among the male elderly was lower than that among the female elderly [2],[3]. The world's average life expectancy extended from 48 years old (1950–1955) to 68 years old (2005–2010), 76 years old (2045–2050) and 82 years old (2095–2100), and was higher in developed countries than in developing ones [1],[4]. The life expectancy has changed over time, by geographical region and nation. According to the World Bank's statistics, the highest life expectancy in 2013 belonged to Japan (83 years old) as opposed to 79 years old in the USA and 76 years old in Vietnam, and Botswana had the lowest life expectancy (47 years old) [5].

Mortality rates exert profound effects on the population scope and structure. A great variety of factors that may affect these rates include age, gender, residence, marital status, living conditions and genetic traits [6]–[11]. Research on mortality rates helps policy makers offer socio-economic resolutions and public health programs in order to reduce mortality rates, prolong life expectancy and lay the foundation for social insurance policies. Vietnam is facing the challenge of population aging in the coming years. According to statistical calculations Vietnam will enter "population aging" from 2017. There is limited knowledge the factors associated with mortality in older people living in Vietnam. Therefore, this study was taken to have scientific evidences for making appropriate recommendations to improve health care for elderly people in the locality. In this article, we used secondary data from the epidemiological population surveillance system in a town of Hai Duong province, Vietnam, with the aim to (1) Analyze the patterns of mortality among the elderly in Chi Linh, Hai Duong during the 2004–2012 period and (2) Identifying some factors associated with the mortality in the elderly.

## 2. Materials and Methods

### 2.1. Data Sources

The study used the secondary date from DESS-Chililab dataset. Besides, the indicators relating to mortality rates of reference groups employed for indirect standardizations were extracted from (1) the sample mortality surveillance system in Vietnam, 2009; (2) the age-specific and gender-specific Canadian mortality data 2009 and (3) The age-specific and gender-specific mortality data in the USA in 2009.

The epidemiological and population surveillance system was established by Hanoi school of Public Health in 2004 in seven communes/towns of Chi Linh rural district, Hai Duong province. Data were collected through this system consisted of basic demographic ones including birth, rate, migration, economic conditions, or marital status.

The sample-based mortality surveillance system in Vietnam 2009 was conducted on 2.6 million people from 668,142 households residing in 192 communes/towns, Vietnam (accounting for 3% of the national population). Data collection and primary findings of the survey were described in detail elsewhere [12]. The dataset of the survey is in the public domain and can be accessed via the GSO's website.

Canada's mortality rates in 2009 [13], the age-specific mortality rates of Canada population by age group and sex, 2009 can be accessed via the Statistics Canada website.

United State of America's mortality rates in 2009 [14], is in the public domain and can be accessed via the CDC's website.

### 2.2. Study Design

This was a longitudinal study; the dataset from DESS of Chililab was used. Main surveys are conducted every two years, while additional ones are carried out every six months for the sake of information update. Data are managed in Structured Query Language (SQL) server and stored in Hanoi school of Public Health.

### 2.3. Measures

#### 2.3.1. Outcome Variables

Time to event: the time from the start of following till the occurrence of an event (death) or, for those who remained alive, till the end of the study (2012). For elderly people moving out of the study site, the end of follow-up period is the point of time when they move out of the study site. Two points of time when the elderly were followed are: the year 2004 (elderly aged 60 or over at this time) and any year after when a person turned 60 years old. Time to event is calculated in months.

Event/death: a binomial variable indicating whether a study subject (an elderly person) died or remained alive during the follow-up period.

#### 2.3.2. Explanatory Variables

Basic demographic variables of the elderly people in the DESS-dataset consist of age, gender, education, marital status and economic conditions.

The ages of the studied elderly people were calculated by subtracting the last follow-up dates (or dates of death) from their dates of birth and then dividing the differences by 365. We assigned participants to 5- year age groups.

Education of participants was categorized into 4 groups: (1) Primary school and under; (2) secondary school; (3) high school and (4) higher than high school.

Union status was categorized into 3 groups: (1) never married; (2) Currently married/living as married and (3) were separated/divorced/windowed.

Socioeconomic status (SES): A household wealth status variable captured underlying long-term wealth based on ownership of consumer goods, dwelling characteristics, and water and sanitation. Principal component analysis was used to derive wealth scores, which were used to rank households into wealth quintiles from the poorest to the wealthiest.

#### 2.3.3. Reference Indicators

The indicators used for indirect standardization were extracted from Vietnam's death-related data in (1) the sample mortality surveillance system in Vietnam, 2009; (2) the age-specific and gender-specific Canadian mortality data 2009 and (3) The age-specific and gender-specific mortality data in the USA in 2009.

### 2.4. Data Analysis

We extracted data from DESS database and exported to STATA 13.0 for analysis. Descriptive analyses were employed to produce mean values, deviations, frequencies and percentages relevant to variables.

Kaplan-Meier Survival analyses and Cox proportional-hazards regression models with Cox Hazard Ratios (CHR) were used to describe the tendencies and mortality patterns as well as analyze some factors associated with mortality. Cox regressions described crude and adjusted association between time to event and some explanatory variables.

In indirect standardization, reference groups were used to compare the mortality in Chililab with that in other groups. Indirect standardization is a method in which small-size samples were used, thereby not ensuring the accuracy of the age-specific and gender-specific ratios. Data important for indirect standardization entail: (1) age- and gender-specific distribution + the number of deaths; (2) age- and gender-specific ratios and death ratios. With this method, we applied standard population ratios to the study sample (Chililab population) to obtain standardized mortality rate and the age- and gender-specific ratios.

### 2.5. Ethical Considerations

The study was approved by Hanoi school of Public Health (HSPH). Besides, the use of the secondary dataset was permitted by HSPH—the institution responsible for keeping the copyright of Chililab's data during 2004–2014. This manuscript has been based on secondary datasets with all identifying information removed

## 3. Results

**Figure 1.**
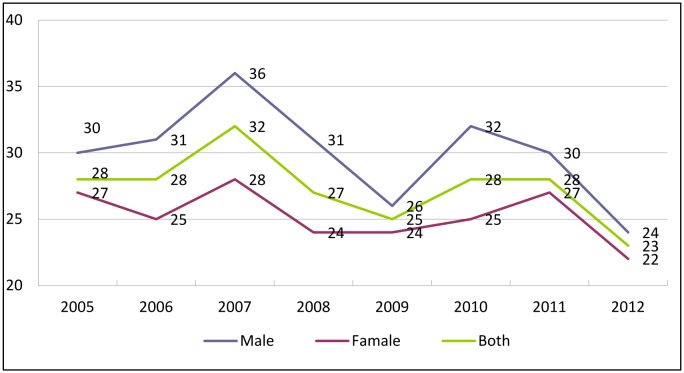
Mortality rate among elderly people (/1000) in Chi Linh, Hai Duong by sex, period 2004–2012.

The mortality rate (MR) of the elderly in this study was inclined to drop between 2005 and 2012; the highest rate was found in 2007 (28/1,000 elderly people) and lowest in 2012 with 22/1,000 elderly people (Figure 1). During the period, the MR in the male elderly group exceeded that in the female one.

**Figure 2.**
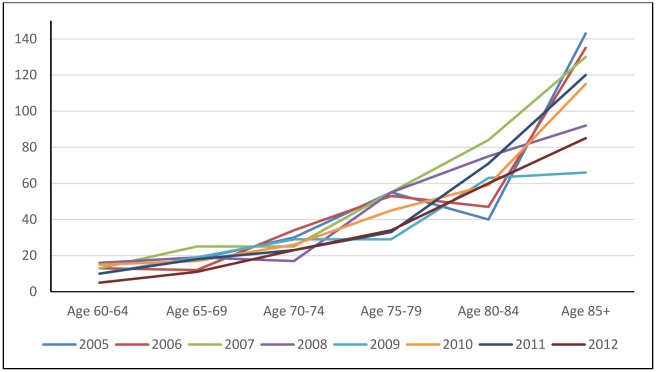
The age-specific mortality rate of male elderly aged 60 or over (per 1,000 elderly people) in Chililab, 2005–2012.

**Figure 3.**
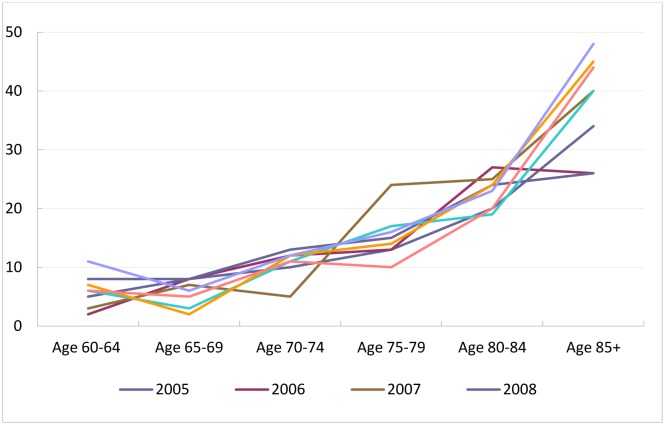
The age-specific mortality rate of female elderly aged 60 or over (per 1,000 elderly people) in Chililab, 2005–2012.

In both the male and female elderly groups, the age-specific mortality rates increased by age group, which means the higher the age group the higher the mortality rate (Figure 2 & 3). In particular, the mortality rate of male elderly people aged 85 years old or reached up to 140/1,000 compared to a lower rate of 50/1,000 among female ones. The increase in the age-specific mortality rate among male elderly people (Figure 2) surpassed that among female ones (Figure 3).

The indirect standardization was employed with Vietnam's data on elderly mortality in 2009 [12], Canada's mortality data in 2009 [13] and that of the USA in 2009 [14]. Table 1 presents the current status of mortality among the elderly group in Chililab in comparison with the mortality rates in Vietnam, Canada and the USA.

The gender-standardized number and mortality rate of elderly deaths in Chililab were smaller than the national figures. The standardized mortality rates stood at 0.75%—the mortality rate among the elderly people in Chililab was 75% of the national one.

Table 1 shows the mortality rate among male elderly people in Chililab standardized by the Canadian mortality rate of 0.82; hence, the MR of male elderly people in Chililab is also lower (82% as much as) that of the Canadian MR. However, in the under 75 age group, the MR among the male elderly people in Chililab is in excess of the Canadian MR, while in the group aged 75 or over, it is lower. With regard to the female elderly, the MR in Chililab equated to the Canadian figure in all but the group aged 85 or over. Compared to the MR among elderly people aged 65 or over in the USA in 2009, that among those in Chililab is lower. It is only equal to 73% of the American figure.

**Table 1.** Standardized mortality rate among elderly people in Chi Linh, Hai Duong, 2009 ^1^.

Gender- age group		Mortality rate among Vietnam elderly people 2009^2^	Mortality rate among Canada elderly people 2009^3^	Mortality rate among America elderly people 2009^4^	Mortality in Chi Linh 2009			Estimated number of death based on Vietnam MR	Estimated number of death based on Canada MR	Estimated number of death based on America MR ^5^
					Elderly population	Number of death	Mortality rate			
**Male**										
Age 60–64		21.84	9.6	–	835	8	9.58	45.69	8.02	–
Age 65–69			15.7	22.73	668	13	19.46		10.49	28.57
Age 70–74			24.5		589	17	28.86		14.43	
Age 75–79		81.79	41.6	56.88	448	13	29.02	69.76	18.64	39.08
Age 80–84			70.0		239	15	62.76		16.73	
Age 85 +			164.5	153.96	166	11	66.27		27.30	25.56
**Female**										
Age 60–64		11.88	6.2	–	1,069	6	5.61	29.74	6.63	–
Age 65–69			9.4	15.26	776	3	3.87		7.29	21.90
Age 70–74			15.8		659	11	16.69		10.41	
Age 75–79		57.45	26.3	41.34	651	17	26.11	84.166	17.12	43.8
Age 80–84			47.4		409	19	46.45		19.39	
Age 85 +			131.8	132.04	405	40	98.77		53.36	59.42
**Total**		**32.43**	**–**	**–**	**6,914**	**173**		**229.356**	**209.80**	**218.33** ^1,2^
**Standardized Mortality Ratio-SMR**								**0.75**	**0.82**	**0.73**

^1^ Applying indirect standardization to compare death-related data in Chililab with those in some reference populations, including Vietnamese national and Canadian elderly people in 2009.

^2^ Sources: Vietnam's sample-based mortality surveillance system in, 2009.

^3^ Canadian age- and gender-specific death rates in 2009: *http://www.statcan.gc.ca/pub/91-209-x/2013001/article/11785/tbl/tbl02-eng.htm*.

^4^ American age- and gender-specific death rates in 2009: *http://www.cdc.gov/nchs/data/nvsr/nvsr60/nvsr60_04.pdf*.

^5^ Age group of 60-64 years old is excluded.

**Table 2.** The associated factors with mortality among elderly people in Chi Linh, Hai Duong, and period 2005–2012 based on the Cox Hazard ratios-HR.

Factors		Crude HR (95% CI)	Adjusted ** HR (95% CI)
***Gender (Reference group: Male)***			
Female		0.69 (0.62–0.75)	0.69 (0.61–0.78)
Age		1.12 (1.11–1.12)	1.10 (1.09–1.11)
***Education (Reference group: primary school and under)***			
Secondary school		0.93 (0.79–1.09)	0.89 (0.76–1.06)
High school		0.91 (0.76–1.06)	0.98 (0.80–1.21)
Higher than high school		1.80 (1.53–2.12)	2.39 (1.93–2.97)
***Married (Reference group: Currently married/living as married)***			
Separated/divorced/windowed		1.33 (1.19–1.49)	1.18 (1.03–1.33))
Never married		1.30 (0.78–2.17)	1.73 (1.03–2.90)
***Occupation (Reference group: Famer)***			
Retired		0.93 (0.74–1.16)	0.58 (0.45–0.74)
Business		0.80 (0.51–1.24)	0.77 (0.49–1.20)
Others		1.09 (0.88–1.35)	1.16 (0.94–1.45)
***Economy (Reference group: poorest)***			
Near-poor (the second)		0.83 (0.72–0.97)	0.85 (0.73–0.99)
Average (the third)		0.95 (0.82–1.10)	1.00 (0.85–1.16)
Middle income (the fourth)		0.92 (0.79–1.08)	0.96 (0.81–1.13)
Wealthy		0.86 (0.74–1.01)	0.89 (0.75–1.11)

In the multivariate analysis using Cox proportional-hazards regression, the female elderly people faced a risk of mortality 69% lower than their male counterparts (*p* < 0.01) when other factors remained the same. Apart from age, marital status and occupation, elderly people with high school education or higher had a higher risk of death (2.4 times) than those with lower education.

Table 2 indicates that as an elderly person lived one more year, his or her risk of death was 10 per cent higher. Separated/widowed/divorced elderly people had a risk of death 1.18 times higher than those living with their spouses and those who were single had a risk of death 1.73 times higher than those living with their spouses when all other variables were adjusted to be the same. Retired elderly people had a lower risk of death than those who engaged in farming (*p* < 0.01), and those from near-poor households faced a 0.85 per cent risk of death when compared to those from poor ones (*p* < 0.05) when all of the other variables were adjusted to be the same.

## 4. Discussion

### 4.1. Mortality in the Elderly over Time

Similar to the world's tendencies [2],[3],[7],[15], the age-specific MR among the elderly people in Chi Linh had a proclivity to go down over the past time. The MR in almost all of the age groups were inclined to decrease thanks to improved health services, technological advancements, as well as the launch of more preventive and interventional programs with promising results. Besides, life expectancy has increased and age-specific mortality rates have reduced as a result of better economic conditions.

Reduction in the crude death rate is never an easy task due to such factors as environmental, economic, cultural, social and technological conditions. Over the past time, the investment in medical care and public health care originated from the national budget as well as other organizations and financial aids have significantly reduced the mortality rate in Vietnam and therefore Vietnam's average life expectancy has increased.

### 4.2. Standardized Mortality Rate in Chi Linh from Some Reference Groups

In order to compare mortality rates across countries or regions with similar population size and socio-economic conditions, researchers employ direct and indirect standardization methods. In our study, we used the latter method with data on Vietnamese mortality rates of elderly people in 2009 [12], Canada's mortality rates in 2009 [13] and the USA's mortality rates in 2009 [14]. Vietnamese, American and Canadian figures were used as those of standard populations. Compared to the number of deaths among Vietnamese elderly people, that among Chililab's elderly group was lower (the mortality rate in Chililab was also lower) when standardizing for both the male and female groups. The standardized mortality rate was 0.75—the MR among elderly people in Chililab was 75 per cent of Vietnamese MR. Chi Linh is a semi-mountainous, well-off town with good living conditions and economy. It is located in Hai Duong—a province on the top list of cities and provinces with fast economic growth and high living standard in Vietnam [16].

### 4.3. Factors Associated with Mortality of the Elderly People in Chililab

The research team employed the Survival analysis technique and presented results of Cox regression analyses on some factors attributable to the mortality of the elderly people in Chililab through calculation of Cox Hazard Ratios (CHR).

***Age:*** In elderly people, the leading cause of death refers to age; as an elderly person lived one more year, his or her risk of death was 10 per cent higher which noticeably rapidly increased in the group aged 75 or over. The older an age group, the higher its MR. The increase in the age-specific MR of the male elderly people was higher than that of female ones. This is also the overall trend of mortality in the elderly throughout the world [6],[7],[13],[14],[17] as well as those in Vietnam [12]. As a person grows older, he or she tends to have compromised immune systems, low resilience, poor health and higher susceptibility to diseases. Even in the case of a person without a specific disease, his or her body parts will age sooner or later, leading to dis-function and eventually fatality (due to ageing). Generally, as the population ages, we will have to confront with numerous challenges, including the “double burden of diseases” [18] as do developing countries: for one thing, we have to keep on coping with infectious diseases, malnutrition and pregnancy complications; and for the other thing, we are challenged by the rapid growth of non-communicable diseases as the health care system is still of poor quality.

***Gender:*** In all age groups, the MR of male elderly people was always higher than that of female ones, which resembles the results of other studies [2]. The difference between two gender groups in terms of MR results from the fact that men and women have different lifestyles. For example, men consume harmful and addictive products such as tobacco, alcohol/beer or illicit drugs more than women, thus suffering from detrimental health impacts as a consequence. That causes men to face a higher risk of diseases than women, which was also indicated in the results of analyzing disease-specific MR in the population and housing census 2009 [19]; the rate in men was 6.6/1,000 people as opposed to 4.6/1,000 people in women. Some studies also proved that the MR among the elderly smokers was higher than elderly non-smokers [6],[20] and in Vietnam a great majority of elderly smokers were men.

***Marital status:*** Marital status is also associated with the risk of death. Separated/divorced/widowed elderly people had the lower probability of survival over time compared to those living alone or with their spouses. In general, living with spouses exerted beneficial impacts on health in the elderly people because it provides with support and social security. Those living with their spouses rarely become stressed and have healthier lifestyles and reduced stress, thereby lessening the stimulation of endocrinal systems and slowing down the progression of atherosclerosis and other diseases [21]. Evidence from a prior retrospective study shows that loosen social relationships and loneliness have associations with the increased risk of death for both men and women [21]. Besides, elderly people who did not live with their spouses were more likely to take up harmful habits (e.g. smoking, alcohol consumption, or physical inactivity) than those living with their spouses [9]; these harmful habits make primary contributions to death in the elderly people.

***Education:*** Elderly people's education was related to their risk of death. Accordingly, those with high levels of education had lower risk than those with lower education. Those with higher education had certain social statuses, succeeded in their careers, and had better jobs and social relationships. They often had a higher income than those with lower education. Therefore, they had better quality of life and lower risk of death. Besides, those with high education had better knowledge and better awareness of their health. They leaded a healthier life and avoided bad health habits; thus, their risk of death was lower. This is not the result of changes when they get older, but a long-term process during which they practiced a healthy lifestyle.

***Household economy:*** Elderly people from well-off households had lower risk of death than those from poor or near-poor households when adjusting for the differences by age, gender and other factors. Those from good economic conditions experienced better daily life and had better opportunity to take care of their own health. Furthermore, they had better access to health care of high quality as they had better economic conditions, i.e. they had better and more suitable choices of health services.

***Living areas:*** Elderly living in urban areas had lower risk of death than those in rural areas. Indeed, many rural residents aged 60 or older still had to work hard on farms and do not have time for relaxation and self-care. Elderly people in urban areas are primarily civil servants or used to do jobs that allowed them to have more free time to relax and take care of their health and had better income. Apart from that, services and facilities for health care in urban areas are of better quality than in the rural areas, and nutritional status of elderly people here are also higher.

## 5. Limitations

The study used secondary data, resulting in certain inevitable limitations. Firstly, factors considered as the causes of death such as health services, health care, supporting services and social security were not mentioned in the study because of data deficiency. Besides, information about the situation of diseases among elderly people is not available. That is the reason why it was not mentioned in this study.

We were not able to access age-specific and gender-specific mortality data of more updated reference groups, but only compared the data in 2009 with those in some populations.

## 6. Conclusion

The study results show that the age-specific mortality rates among elderly people living in Chi Linh had an inclination to decline over time. The MR among male elderly people has always been higher than that among female ones in all age groups. Mortality among elderly people in Chililab in 2009 was lower than Vietnamese, Canadian and American figures after applying the indirect standardization method based on reference groups. Some factors relating to mortality among elderly people included education, economic conditions and living with a spouse and age.

The study results suggested Elderly people supporting programs be focused on elderly people who lived alone, had low education or lived in low-income households. Elderly people are considered a vulnerable group in terms of access to health care programs or services, i.e. they had limited access to such programs or services.

## References

[1] United Nations, Department of Economic and Social Affairs, Population Division (2010). World Population Prospects. The 2010 Revision.

[2] Wang H, Dwyer-Lindgren L, Lofgren KT (2012). Age-specific and sex-specific mortality in 187 countries, 1970–2010: a systematic analysis for the Global Burden of Disease Study 2010. Lancet.

[3] Christensen K, Doblhammer G, Rau R (2009). Ageing populations: the challenges ahead. Lancet.

[4] United Nations, Department of Economic and Social Affairs, Population Division (2012). World Population Prospects. The 2012 Revision.

[5] The World Bank Life Expectancy at Birth.

[6] Gillum RF, Obisesan TO (2010). Physical activity, cognitive function, and mortality in a US national cohort. Ann Epidemiol.

[7] Gomez-Olive FX, Thorogood M, Bocquier P (2014). Social conditions and disability related to the mortality of older people in rural South Africa. Int J Epidemiol.

[8] Holt-Lunstad J, Smith TB, Layton JB (2010). Social relationships and mortality risk: a meta-analytic review. PLoS Med.

[9] Ikeda A, Iso H, Toyoshima H (2007). Marital status and mortality among Japanese men and women: the Japan Collaborative Cohort Study. BMC Public Health.

[10] Kandler U, Meisinger C, Baumert J (2007). Living alone is a risk factor for mortality in men but not women from the general population: a prospective cohort study. BMC Public Health.

[11] Rizzuto D, Fratiglioni L (2014). Lifestyle factors related to mortality and survival: a mini-review. Gerontology.

[12] Hoa NP, Rao C, Hoy DG (2012). Mortality measures from sample-based surveillance: evidence of the epidemiological transition in Vietnam. Bull World Health Organization.

[13] Statistics Canada (2009). Mortality: Overview, 2008 and 2009.

[14] Murphy SL, Xu JQ, KD K (2012). Deaths: Preliminary data for 2010. National vital statistics reports. National Center for Health Statistics.

[15] Minino AM, Murphy SL (2012). Death in the United States, 2010. NCHS Data Brief.

[16] General Statistics Office (2009). Results of the Household living standard surveys 2008.

[17] Mostafa G, van Ginneken JK (2000). Trends in and determinants of mortality in the elderly population of Matlab, Bangladesh. Soc Sci Med.

[18] Thang P, Hy DTK (2009). Overview of the policy on elderly care adapting to the changing age structure in Vietnam, 2009.

[19] General Statistics Office (2010). Fertility and mortality levels in Vietnam: Current situation, Trends and Differences, 2010.

[20] Gu D, Kelly TN, Wu X (2009). Mortality attributable to smoking in China. N Engl J Med.

[21] Eng PM, Rimm EB, Fitzmaurice G (2002). Social ties and change in social ties in relation to subsequent total and cause-specific mortality and coronary heart disease incidence in men. Am J Epidemiol.

